# Factors influencing immunologic response to hepatitis B vaccine in adults

**DOI:** 10.1038/srep27251

**Published:** 2016-06-21

**Authors:** Shigui Yang, Guo Tian, Yuanxia Cui, Cheng Ding, Min Deng, Chengbo Yu, Kaijin Xu, Jingjing Ren, Jun Yao, Yiping Li, Qing Cao, Ping Chen, Tiansheng Xie, Chencheng Wang, Bing Wang, Chen Mao, Bing Ruan, Tian’an Jiang, Lanjuan Li

**Affiliations:** 1 State Key Laboratory for Diagnosis and Treatment of Infectious Diseases, Collaborative Innovation Center for Diagnosis and Treatment of Infectious Diseases, The First Affiliated Hospital, College of Medicine, Zhejiang University, Hangzhou 310003, China; 2 Zhejiang Provincial Center for Disease Control and Prevention, Hangzhou 310051, China; 3 Zhejiang Institute of Medical-care Information Technology, Hangzhou 311112, China; 4 Division of Epidemiology, The Jockey Club School of Public Health and Primary Care, Hong Kong, The Chinese University of Hong Kong, China; 5 Shenzhen Municipal Key Laboratory for Health Risk Analysis, Shenzhen Research Institute of The Chinese University of Hong Kong Shenzhen, Guangdong ProvinceChina; 6 Department of Ultrasound, First Affiliated Hospital, College of Medicine, Zhejiang University, Hangzhou 310003, China

## Abstract

Hepatitis B was still a worldwide health problem. This study aimed to conducted a systematic review and meta-analysis to assess a more precise estimation of factors that influence the response to hepatitis B vaccine in adults. Our included studies examined seroprotection rates close to the end of vaccination schedules in healthy adult populations. This meta-analysis including 21053 adults in 37 articles showed that a significantly decreased response to hepatitis B vaccine appeared in adults (age ≥ 40) (RR:1.86, 95% CI:1.55–2.23), male adults (RR:1.40, 95% CI:1.22–1.61), BMI ≥ 25 adults (RR:1.56, 95% CI:1.12–2.17), smoker (RR:1.53, 95% CI:1.21–1.93), and adults with concomitant disease (RR:1.39, 95% CI:1.04–1.86). Meanwhile, we further found a decreased response to hepatitis B vaccine appeared in adults (age ≥ 30) (RR:1.77, 95% CI:1.48–2.10), and adults (age ≥ 60) (RR:1.30, 95% CI:1.01–1.68). However, there were no difference in response to hepatitis B vaccine both in alcoholic (RR:0.90, 95% CI:0.64–1.26) and 0-1-12 vs. 0-1-6 vaccination schedule (RR:1.39, 95% CI:0.41–4.67). Pooling of these studies recommended the sooner the better for adult hepatitis B vaccine strategy. More vaccine doses, supplemental/additional strengthening immunity should be emphasized on the susceptible population of increasing aged, male, BMI ≥ 25, smoking and concomitant disease. The conventional 0-1-6 vaccination schedule could be still worth to be recommended.

Hepatitis B as an acute and chronic communicable disease, has been a worldwide health problem estimated to lead to between 500,000 to 1.2 million deaths every year through causing chronic hepatitis, cirrhosis and hepatocellular carcinoma^1^. The prevalence of HBV infection varies significantly in different areas: prevalence of chronic infection with HBV estimates range between 0.1–0.7% in Western, Northern, and Central Europe, while those considerably higher in Eastern and Southern European countries, such as Italy (0.2–4.3%), Turkey (2.5–9%), and Romania (5.6%)^2^,^3^. In Alaska, 41% had anti-HBs levels of >10 mIU/ml 7 to 9 years after booster vaccination at birth^4^, even 51% had this protective levels 30 years after receiving the primary series without subsequent doses in Alaska native persons^5^. In China, the HBsAg carrier rate was 8.75% in 1979, 9.75% in 1992, and 7.18% in 2006^6^; in Taiwan, the values are as high as 15–20% in adults^7^; and in the Middle East and North Africa region, the HBV infection estimates are various such as 9.8% in Egypt, 7.4% in Iran, 2.4% in Lebanon and 6.9% Libya from the prisoners; 50.7% in Iran, 8.6% in Israel, 2.8% in Lebanon, 4.5% in Libya, 2.6% in Palestine, 6.1% in Saudi Arabia from the injecting drug users^8^. In Gambia, 13.2% were found to carry HbsAg^9^ and national infant HBV vaccination controlling chronic infection had 94% vaccine efficacy^10^. HBV can be transmitted in many ways, with sexual intercourse and mother-to-child transmission being the most common. Between 15% and 40% of those infected develop acute or chronic liver disease and liver failure, cirrhosis or hepatocellular carcinoma may result.

Many countries have gradually adopted the HBV vaccine in national immunization programs since the World Health Organization (WHO) recommended vaccination for children in 1990s. Most individuals with chronic hepatitis B are asymptomatic and therefore ignorant of their infection status but HBV vaccination, if used for primary prevention, can significantly lower the risk of infection. HBV vaccination triggers antibody response and antibody to hepatitis B surface antigen (anti-HBs) levels ≥10 IU/L are usually regarded as seroprotection for most vaccinees.

Vaccination efficacy among children has been widely studied, but there remains a large proportion of adult populations who are as yet unvaccinated. A previous meta-analysis in 2002 observed many factors influencing response to hepatitis B vaccine, especially a decrease response to recombinant HBV vaccine at higher ages^11^, which suggested that earlier vaccination should be prioritized for prevention at the population level. However, in the last decade, numerous emerging reports, which focused on the seroprotection rate of hepatitis B vaccine in adults^12^,^13^,^14^,^15^,^16^,^17^,^18^,^19^,^20^,^21^,^22^,^23^,^24^,^25^,^26^,^27^,^28^,^29^,^30^,^31^,^32^,^33^,^34^,^35^,^36^,^37^,^38^,^39^,^40^,^41^,^42^,^43^,^44^,^45^,^46^,^47^,^48^, are still inconclusive to immunize what adults are the most appropriate in order to increase the seroprotection rate. Factors influencing immunologic response to hepatitis B vaccine in adults have been inconsistently examined in existing studies. In this study, we conducted a systematic review and meta-analysis to update and assess a more precise estimation of factors that influence the response to HBV vaccine.

## Material and Methods

### Search strategy

To find all relevant publications that investigated the association between adult and hepatitis B vaccine and seroprotection, a systematic literature search was independently conducted by two individual investigators with the same method in PubMed, Embase and Cochrane Library using the keywords “hepatitis B vaccine”, “HBV”, “adult”, “anti-HBs” were used. Data were collected from the full-published paper and no language or race restriction was used. Bibliographies of relevant review articles were also screened to supplement the electronic searches.

### Inclusion criteria

Included studies met the following criteria (1) original research papers; (2) prospective or retrospective studies, including cohorts and trials; (3) sample size ≥10; (4) healthy subjects, pregnant women, participants with diabetes, chronic renal failure or other diseases but without congestive hepatopathy or infectious diseases; (5) mean sample population age ≥18 years; (6) populations are largely vaccine naive; (7) seroprotection (generally defined as antibody-HBs at a titer of >10 mlU/mL) is assessed at least one month after last recombinant vaccine dose in the majority of participants.

### Article screening

Citations were electronically downloaded into reference management software and duplicate citations were electronically/manually excluded. Where studies had multiple reports, the most recent or most complete article was retained. The remaining citations were screened independently by two reviewers using pre-defined criteria. Full-text versions of potentially relevant citations were obtained and again screened independently by two reviewers according to pre-defined criteria. Disagreement was resolved by the opinion of a third reviewer.

### Data extraction and quality assessment

The data was independently extracted and then cross-checked by two investigators according to a standard format as follows: author, publication year, age, country, male/female participants, body mass index (BMI), vaccination schedule, time of immunological assessment after last vaccine dose, vaccine characteristics and injection pathwayIf necessary data were unavailable in articles, a request was sent to the author for relevant data. The definition of age strata varied among studies. We considered age ≥16 years as adult and age ≥40 years as a default definition of older age for study participants. We considered individuals with anti-HBs titers ≥10 IU/L to be seroprotective after completion of vaccination against HBV. In a few articles, those data are also available if the seroconversion was defined as anti-HBs titers ≥10 IU/L. The articles were divided into four quality levels such as high, moderate, low, and very low by GRADE evidence profile, which allocates original ranks of low score to observational studies and high score to RCTs^49^.

### Statistical analysis

In this meta-analysis, we calculated the relative risks (RRs) and 95% confidence intervals (CIs) by comparing the valid and invalid participators in the experimental group and control group of recruited articles. Statistical heterogeneity in the studies was examined by the Q statistic. We evaluated the heterogeneity in these studies by this method, *I*^*2*^ = 100 %*(Q-df)/Q. A fixed-effect model was used to analyze the data if there was no statistical difference of heterogeneity (p ≥ 0.05). Otherwise, a random-effect model would be selected.

Subgroups analyses were defined in advance/defined according to the reported data, and studies or results were grouped according to age (older or younger than 40), sex, smoking status, alcoholism, vaccine administration (0-1-12/0-1-6 vaccination schedule), geographical location (Asians/Non-Asian). Sensitivity analysis was performed to estimate the stability of the model by removing each study in turn. Additionally, publication bias was assessed through the funnel plot and Egger’s linear regression test^50^. All statistical analyses were conducted by Stata 12.0 software.

## Results

### Characteristics of eligible studies

**Figure 1:**
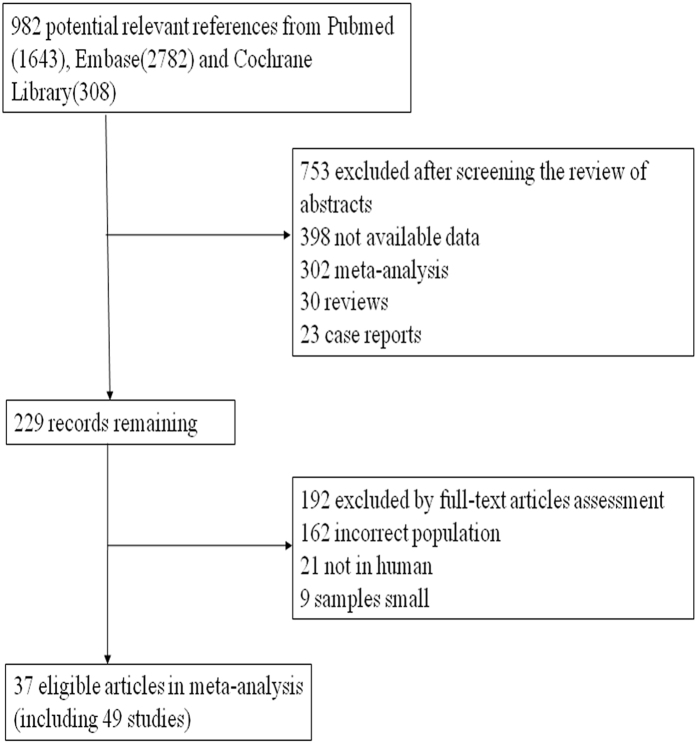
Flow diagram of the study selection process.

We finally identified 21053 adults from 37 articles up to June 30, 2015 through electronic and manual searches (Fig. 1). Nine hundred and forty-six studies were excluded according to the mentioned criteria. The characteristics of included studies for this meta-analysis are presented in Table 1 and the majority of the studies were assessed as being of good quality (Table 2).

The studies were largely prospective cohort (n = 5), retrospective cohorts (n = 23), or randomized trials (n = 10). Studies varied considerably in size and were conducted among many countries. The three vaccine doses tended to be administered either at months 0, 1, 6 or 0, 1, 12 and recombinant vaccine doses ranged from 10 μg to 40 μg.

### Meta-analysis results

#### Heterogeneity test result and subgroup analysis

**Figure 2:**
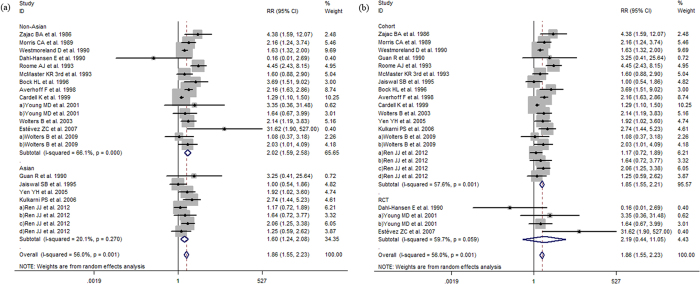
(**a**) The relative risks of response to HBV vaccine between adults age ≥40 and adults age <40. (**b**) The relative risks of response to HBV vaccine between adults age ≥40 and adults age <40. Comparing with adults age <40, the RRs show decreased response to HBV vaccine among adults age ≥40 in cohort and overall studies.

**Figure 3:**
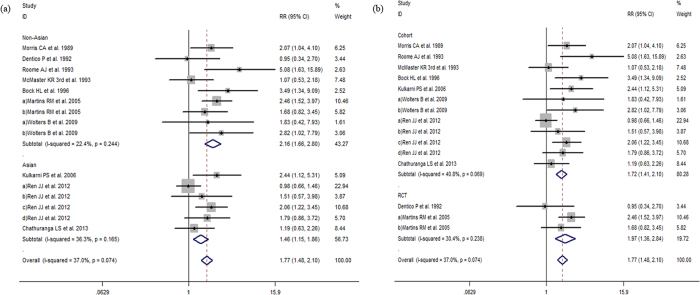
(**a**) The relative risks of response to HBV vaccine between adults age ≥30 and adults age <30. (**b**) The relative risks of response to HBV vaccine between adults age ≥30 and adults age <30 grouped by study design. Comparing with adults age <30, the RRs indicate reduced response to HBV vaccine among adults age ≥30 both in cohort and RCT studies.

**Figure 4:**
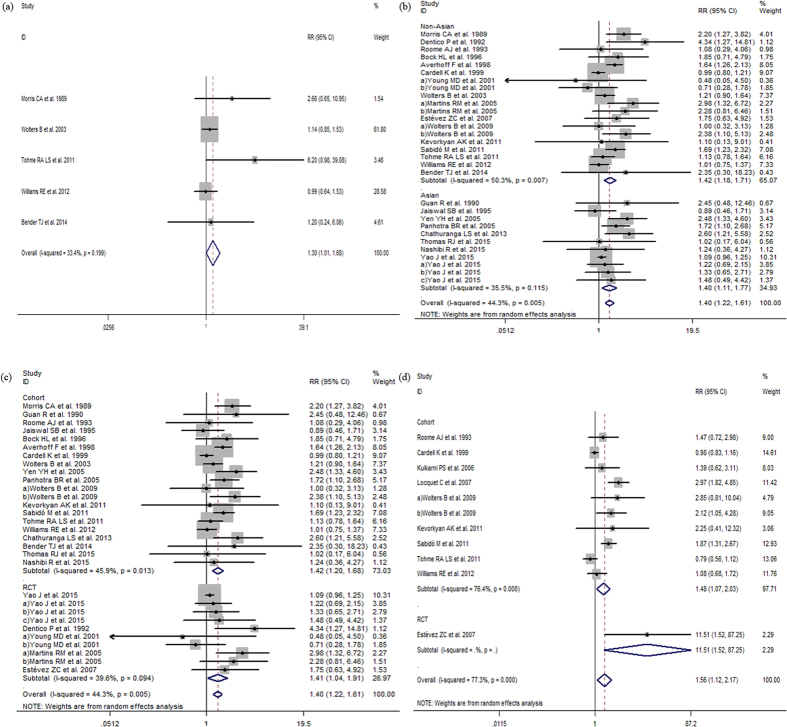
(**a**) The relative risks of response to HBV vaccine between adults age ≥60 and adults age <60. (**b**) The relative risks of response to HBV vaccine between male adults and female adults. (**c**) The relative risks of response to HBV vaccine between male adults and female adults grouped by study design. Comparing with female adults, The RRs suggest declined response to HBV vaccine among male adults both in cohort and RCT studies. (**d**) The relative risks of response to HBV vaccine between BMI ≥25 adults and BMI <25 adults.

**Figure 5:**
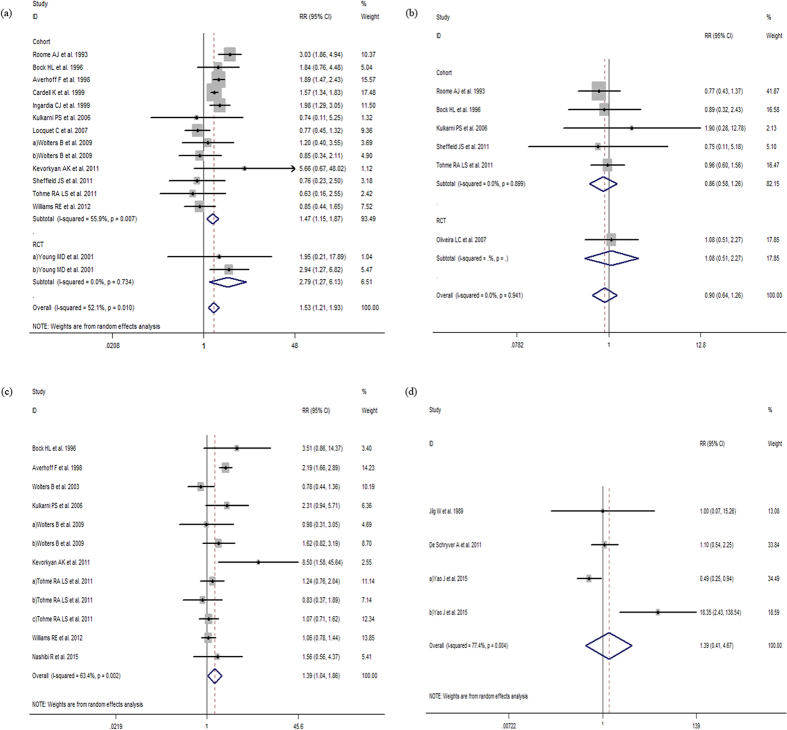
(**a**) The relative risks of response to HBV vaccine between smoker and nonsmoker. (**b**) The relative risks of response to HBV vaccine between alcoholic and nonalcoholic. (**c**) The relative risk of response to HBV vaccine between adults with concomitant disease and healthy adults. (**d**) The relative risk of response to HBV vaccine between 0-1-12 and 0-1-6 vaccination schedule.

The *Q*-tests of heterogeneity were marked in partial groups and then the pooled RRs were calculated by the random-effect models and fixed-effect models. Meta-analysis revealed that vaccine non-response rates were significantly greater in older participants (age ≥40 vs. <40 years, RR:1.86, 95% CI:1.55 to 2.23, *I*^*2*^ = 56%, *P*  = 0.001; age ≥30 vs. <30 years, RR:1.77, 95% CI:1.48 to 2.10, *I*^*2*^ = 37%, *P*  = 0.074; age ≥60 vs. age<60 years RR:1.30, 95% CI:1.01 to 1.68 *I*^*2*^ = 33.4%, *P*  = 0.199). Non-response was also more likely among males (male adults vs. female adults, RR:1.40, 95% CI:1.22 to 1.61, *I*^*2*^ = 44.3%, *P*  = 0.005); overweight participants (BMI ≥ 25 adults vs. <25, RR:1.56, 95% CI:1.12 to 2.17, *I*^*2*^ = 77.3%, *P* < 0.001); smokers (smoker vs. nonsmoker, RR:1. 53, 95% CI:1.21 to 1.93, *I*^*2*^ = 52.1%, *P*  = 0.01) and those with concomitant disease compared to healthy participants (RR:1.39, 95% CI:1.04 to 1.86, *I*^*2*^ = 63.4%, *P*  = 0.002) (Figs 2a, 3a, 4a,b,d and 5a,d). However, there were no differences in response to HBV by alcoholic status (alcoholic vs. nonalcoholic, RR:0.90, 95% CI:0.64 to 1.26, *I*^*2*^ = 0, *P*  = 0.941) or vaccination schedule (vaccine delivered at months 0-1-12 vs. 0-1-6, RR:1.39, 95% CI:0.41 to 4.67, *I*^*2*^ = 77.4%, *P*  = 0.004).

Subgroup analysis by study location and age indicates that older adults (≥40 years) from non-Asian countries revealed that in contrast with Asians, specially non-Asians in older adults (age ≥ 40) may be slightly less response to hepatitis B vaccine than younger adults (age < 40) (RR:2.02, 95% CI:1.59–2.58; RR:1.60, 95% CI:1.24–2.08), consistent with the region result of older adults (age ≥ 30) (RR:2.16, 95% CI:1.66–2.80; RR:1.46, 95% CI:1.15–1.86). Particularly comparing with Asians, the male in non-Asians has a similar nonresponse to females (RR:1.42, 95% CI:1.18–1.71; RR:1.40, 95% CI:1.11–1.77).

When studies were subdivided by study design results were consistent and lower response was seen among studies with older participants and male participants and again, no difference was observed by alcoholic status (Figs 2b, 3b, 4c,d and 5a,b).

#### Sensitivity analysis and publication bias

Sensitivity analysis was conducted to estimate the stability of the results and indicated no significant change if any one study was excluded. Funnel plot asymmetry was assessed by means of Egger’s linear regression test and showed that there was significant publication bias in the following groups: age of 40 years, gender and BMI (age40: *t*  = 2.54, *P*  = 0.019; sex: *t*  = 2.99, *P*  = 0.006; BMI: *t*  = 2.70, *P*  = 0.025).

## Discussion

When stratified by demographic features, our study showed a lower response in older adults (especially age ≥ 40), male adults and overweight adults (BMI ≥ 25), smoker and adults with concomitant disease after completion of vaccination against hepatitis B.

Our study indicated that young adults have a higher seroprotection rate to hepatitis B vaccine than other age groups (age30: RR = 1.77; age40: RR = 1.86; age60: RR = 1.30). It means that the earlier adult vaccination was inoculated at an age, the better efficiency is. The lower responsiveness to hepatitis B vaccines in older adults might result from the waning immunity with age. In previous studies, it did not find a significant association between age and the immune response^43^,^44^. The reason may be that most adults in the study were under the age of 40 years. However, in an observational prospective study of 666 participants, the percentage of nonresponders elevated gradually with age^51^. Another study aligned with our findings also found that younger age and female gender were predictive of better response^52^. It indicated in our study that population in non-Asians were both better in age of 40 or 30 years (age30: RR = 2.16; age40: RR = 2.06). Surprisingly our result also showed that the response rate in the younger adults (age < 60) was better than those older (age ≥ 60), different from the previous study^44^. Some studies reported seroprotection rates of hepatitis B vaccine in older adults (aged ≥ 60 years) range from 30% to 80% and rely on these factors such as study population, vaccination plan, vaccination history and type of vaccine^40^.

Besides age in our study, male gender both in Asians and non-Asians may be associated with nonresponse to hepatitis B vaccine. It may be owing to the opposite effects of sex hormone androgen and estrogen. This difference is experimentally repeated in animal models, which indicated to be activated by sex hormones in genetic regulation. Moreover, there are numerous immunological genes appearing on the X chromosome while few ones are mapped on the Y chromosome. Estrogen activates monocytes to secrete IL-10, which induces IgG and IgM secretion through B-cells in turn^53^, while testosterone damages the production of IgG and IgM from B-lymphocytes, as well as restrains producing IL-6 from monocytes^54^. The hormones’ joint effects on the epigenetic adjustment of genetic expression, and gene structure on the X chromosome differing between XX females and XY males, will partly account for vaccine response heterogeneity in gender^55^. Based on our results, future programme should be emphasized on males both Asians and non-Asians, who tend to have less response to hepatitis B vaccine.

BMI might influence the level of vaccine response^25^. The low response to vaccination of overweight on vaccine could be due to the main distribution of the vaccine in fat not in muscle. This could hinder absorption and enable denaturation of the vaccine antigen by enzymatic action^25^. Another possible interpretation is damaged proliferation and function of the antibody-secreting plasma cells.

The lower immunogenicity of hepatitis B vaccine was linked with smoking and male gender. In smokers, smoking can affect cells and humoral mediated immune responses in humans and animals. Nicotine restrains the antibody-forming cell response by damaging antigen-mediated pathway in T cells and intracellular calcium response. In addition, a high prevalence of HBV markers has been reported in alcoholics. Persistent alcohol intake could restrain immune responses especially in female^56^. But some studies also reported that difference was undetected between alcohol consumption and seroprotection of hepatitis B vaccination^31^,^38^. In this study, the inapparent association within alcoholic subgroup may result from the small sample size and drinking is not common in females.

Aside from those factors, patients with concomitant disease usually have a complicated and inconstant status due to diverse pathogenesis. Although some researches found no association between comorbidity and seroprotection^36^,^37^, comorbidity may be a significant element decreasing the efficacy of hepatitis B vaccine from this analysis and others^23^,^27^, which could bring immunity disturbance. However, the detailed mechanisms between the poor response to hepatitis B vaccine and adults suffering from concomitant disease are still incompletely understood.

What’s more, the four reports regarding different vaccination schedules in adults such as 0-1-6 and 0-1-12 schedules are still controversial^13^,^39^,^47^,^48^. Three studies among them^13^,^39^,^47^ observed no difference in seroconversion rates between these two schedules (as in these cases they were all nearly 100%), while another study^48^ showed a higher seroconversion rate in individuals with the 0-1-12 schedule. Our meta analysis found no difference for seroconversion rate one month after the third injection both in 0-1-6 and 0-1-12 vaccination schedules. Thus in consideration of timing and vaccination compliance, the conventional 0-1-6 vaccination schedule could be still worth to be recommended.

Recently, emerging studies tended to suggest the genetic determinants of heterogeneity in response to the vaccines against hepatitis B. In twins study, 60% of the phenotypic variance was interpreted for the anti-HBs immune response by additive genetic while 40% by non-shared environmental effects^57^. Asians and non-Asians as study location may also play an important role in seroprotection efficiency of hepatitis B vaccine in adults. The percentage of nonresponders after hepatitis B vaccine remarkably varied in ethnic groups, which may result from the difference of environmental surroundings, the mutation rate and genetic variability, especially at the human leucocyte antigen (HLA) genetic region. However, it is also hard to accurately locate the variation affecting the HBV response in the HLA locus as a result of the long-range linkage disequilibrium in this area^58^. It needs further studies to explore.

In a word, the factors mentioned above suggested these factors consisting of elder adults, male, BMI ≥ 25, smoking and concomitant disease would be the significant variables reducing the immune response to hepatitis B vaccination. Those who are more likely to have non-response should be checked for seroprotection level and offered additional booster vaccinations. Thereby finding those without immunization and improving overall immunization rates across the population should be emphasized.

Our results should be interpreted in view of the following limitations. First, publication bias was identified among studies reporting rates by age groups, gender and BMI groups. Second, for the various subgroup analyses, sample size is diminished and therefore CIs are wide leading to less accurate estimates of response. Third, due to differences in lifestyle characteristics in different studies’ population, significant heterogeneity was present in study, even among subgroup estimates. In addition, there were poor reporting in some included studies and limited inclusion in subgroup analyses such as BMI, smoking status, alcohol status and concomitant disease. What’s more, several different vaccines were used in the different studies, which had different immunogenicity. Engerix B (with 20  μg HBsAg per dose) is more immunogenic than Recombivax (with 10 μg per dose), the difference being seen especially in older individuals. Twinrix is similar immunogenicity to Engerix-B. A multi-center study found that a S-PreS1/PreS2-vaccine (Hepacare) is also more immunogenic than Engerix B^2^. Despite limitations, in this work, we systematically sought out all published literature relevant to our research question and then carefully screened studies and extracted data in duplicate using protocols to ensure high quality and consistency in the extracted data. Missing data were sought from authors and studies results were statistically combined to provide robust estimates of the factors associated with poorer immunological response. To our knowledge, this is the first study to examine multiple factors associated with vaccine response and important differences have been found.

## Conclusions

Taken together, this meta-analysis indicated that there were lower seroprotection rates to hepatitis B vaccine in the subgroups of increasing aged adults, male, BMI ≥ 25, smoking and concomitant disease, and more vaccine doses, supplemental/additional strengthening immunity should be focused on this specific population. No difference in seroconversion rates between 0-1-6 and 0-1-12 vaccination schedule was observed, but in consideration of timing and vaccination compliance, the vaccination 0-1-6 schedule could be still worth to be recommended. In order to obtain accurate effectiveness of hepatitis B vaccine in adults, more large-scale studies should be conducted in the future.

## Additional Information

**How to cite this article**: Yang, S. *et al.* Factors influencing immunologic response to hepatitis B vaccine in adults. *Sci. Rep.* **6**, 27251; doi: 10.1038/srep27251 (2016).

## Figures and Tables

**Table 1:** Summary of studies investigating the response to hepatitis B vaccine in adults.

Author	Year	Studydesign	Age (years)	Populationcharacteristics	Country	Male/Female	BMI	Schedule (months)	Follow-up (After last does of vaccine)	Vaccinedetail	Injection pathway	Geometric mean titer (IU/L)	Seroprotection reached n/% (>=10 mlU/mL)
Zajac B. A. *et al.*	1986	Retrospective cohort	20–70	Healthy adults	USA	NA	NA	0-1-6	1–6 months	10 μg recombinant vaccine	IM	a) 300 IU/L (2.5 ug);b) 350 IU/L (5 ug);c) 1250 IU/L (10 ug);d) 1000 IU/L (20 ug)	a) 98% (2.5 ug);b) 89% (5 ug);c) 97% (10 ug);d) 87% (20 ug)
Jilg W. *et al.*	1989	Randomized trials	a) 24.7 ± 2.1 b) 24.4 ± 1.7 c) 24.6 ± 1.8	Healthy medical students	Germany	a) 12/17;b) 16/14;c) 16/14	NA	a) 0-1-2-12; b) 0-1-6; c) 0-1-12	12 months	10 μg recombinant vaccine	IM	a) 53 IU/I (0-1-2);b) 5846 IU/L (0-1-6);c) 19912 IU/L (0-1-12)	Seroconversion rate in all three groups was 100% after the third dose.
Morris C. A. *et al.*	1989	Retrospective cohort	19–60+	Health care volunteers	United Kingdom	79/136	NA	0-1-6	1–2 months	2 μg recombinant vaccine, Engerix-B	ID	NA	80.9%
Westmoreland D. *et al.*	1990	Retrospective cohort	17–71	Occupational risk of infection	United Kingdom	304/1016	NA	0-1-6	6–8 weeks	20 μg recombinant vaccine, Engerix-B	IM	NA	90.50%
Guan R. *et al.*	1990	Retrospective cohort	40 ± 7.7 range: 23–54	Chronic renal failure	Singapore	11/18	NA	0-1-2-6	6 months	40 μg recombinant vaccine, Engerix-B	IM	112 IU/L	69% (>2.1 IU/L, 79%)
Dahl-Hansen E. *et al.*	1990	Randomized trials	21–62	Healthy adults	Norway	30/109	NA	0-1-6	3 months	recombinant vaccine,20 μg Engerix-B and 10 μg Recombivax	IM	a) 189 IU/L (SKR 20 ug);b) 99 IU/L (MSD 10 ug)	100.0%
Dentico P. *et al.*	1992	Randomized trials	18–60	Volunteer employees	Italy	a) 43/57;b) 35/65	NA	0-1-6	1–42 months	a) 10 μg recombinant vaccineb) 20 μg recombinant vaccine	IM	a) 1252 IU/L (10 ug);b) 1340 IU/L (20 ug)	a) 87% (10 ug);b) 97% (20 ug)
Roome A. J. *et al.*	1993	Retrospective cohort	Mean: 39.3 range: 14–74	Healthy adults	USA	510/18	NA	0-1-6	1–6 months	recombinant vaccine, Recombivax HB	NA	235 IU/L	88.1%
McMaster K. R. 3rd *et al.*	1993	Retrospective cohort	NA	Most firefighters	USA	NA	NA	0-1-2-6	1–2 months	2 μg recombinant vaccine, Engerix-B	ID	NA	90.5%
Jaiswal S. B. *et al.*	1995	Retrospective cohort	NA	Chronic renal failure	India	29/11	NA	0-1-6	1 month	40 μg recombinant vaccine, Engerix-B	IM	NA	50.0%
Bock H. L. *et al.*	1996	Prospective cohort	28 ± 10.6	Health care staff and their relatives	Germany	176/704	NA	0-1-6	1 month	20 μg recombinant vaccine, Engerix-B	IM	1989 IU/L	97.8%
Averhoff F. *et al.*	1998	Retrospective cohort	41	Health care workers	USA	1335/416	NA	0-1-6	1 month	recombinant vaccine, 20 μg Engerix-B and 10 μg Recombivax	IM	<40 years of age: a) 2138 IU/L in Engerix-B,b) 1047 IU/L in Recombivax-HB;≥40 years of age: a) 1000 IU/L in Engerix-B,b) 288 IU/L in Recombivax-HB	a) 90% in Engerix-B;b) 86% in Recombivax-HB
Cardell K. *et al.*	1999	Prospective cohort	Mean: 36 range: 19–63	Health care workers	Sweden	239/1167	NA	0-1-6	2 months	2 μg recombinant vaccine, Engerix-B	ID	NA	68.3%
Ingardia C. J. *et al.*	1999	Retrospective cohort	23.8 ± 5.6 range: 15–40	Pregnant women	USA	0/80	27.7 ± 7.0 range: 18–56	0-1-6	11.1 ± 5.1 weeks	20 μg recombinant vaccine, Engerix-B	IM	NA	45.0%
Young M. D. *et al.*	2001	Randomized trials	a) 39.2 range: 18–65 b) 38.8 range: 18–65	Healthy adults	USA	a) 62/90;	NA	0-1-6	3–4 weeks	a) 20 ug recombinant vaccine; Hepacare; b) 20 ug recombinant vaccine; Engerix-B	IM	90% of vaccinees had titers ≥100 IU/L in both groups.	a) 98% in Hepacare;
						b) 60/91							b) 88% in Engerix-B
Wolters B. *et al.*	2003	Retrospective cohort	Mean: 54 range: 17–84	Older adults	Germany	51/53	NA	0-1-6	16.8 months (range 1–36 months)	20 μg recombinant vaccine, Twinrix	IM	NA	46.0%
Martins R. M. *et al.*	2004	Randomized trials	a) 20–30 b) 31–40	Healthy adults	Brazil	a) 364/114; b) 352/134	NA	0-1-6	28–100 days	a) 20 μg recombinant vaccine, Butang;b) 20 μg recombinant vaccine, Engerix-B	IM	a) Butang^®^, 351.1 in newborn infants, 3600.0 in children, 746.3 in adolescents, 453.5 in adults 20–30 years old, and 122.7 in adults 31–40 years old;b) Engerix-B, 1530.6 in newborn infants, 2753.1 in children, 1284.3 in adolescents, 1369.0 in adults 20–30 years old, and 686.2 in adults 31–40 years old	a) Butang, 93.7% in newborn infants, 100% in children, 95.1% in adolescents, 91.8% in adults 20–30 years old, and 79.8% in adults 31–40 years old;b) Engerix-B, 97.5% in newborn infants, 97.7% in children, 96% in adolescents, 95.5% in adults 20–30 years old, and 92.4% in adults 31–40 years old
Yen Y. H. *et al.*	2005	Retrospective cohort	Mean: 36.6 range: 25–70	Health care workers	China	50/200	NA	0-1-6	8 months	20 μg recombinant vaccine, Engerix-B	IM	5 of 8 responders were 10.5, 199.3, 396.9, 822.2 and 1000 IU/L, respectively.	86.4%
Panhotra B. R. *et al.*	2005	Retrospective cohort	34.6 ± 8.2 range: 21–60	Health care workers	Saudi Arabia	620/682	NA	0-1-6	3 months	20 μg recombinant vaccine, Engerix	IM	NA	92.2%
Kulkarni P. S. *et al.*	2006	Prospective cohort	33 ± 8.645	Healthy adults	India	766/22	22.4 ± 2.8	0-1-6	1 month	20 μg recombinant vaccine, Batch	IM	443 IU/L	96.0%
Estévez Z. C. *et al.*	2006	Randomized trials	20–64	Healthy adults	Cuba	167/293	NA	0-1-2	1 month	20 μg recombinant vaccine, Heberbiovac HB	IM	931.18 IU/L	97.0%
Locquet C. *et al.*	2007	Retrospective cohort	35 ± 10.4 range: 17–65	Women healthcare workers	France	0/880	23.4 ± 4.4	a) 0-1-2-12;	1–169 months	20 μg recombinant vaccine, Genhevac Pasteur/20 μg recombinant vaccine, Engerix GlaxoSmithKline	IM	NA	92.0%
								b) 0-1-6					
Sabidó M. *et al.*	2007	Retrospective cohort	33 ± 10.51	Health care workers	Spain	437/1621	23.50 ± 3.76	0-1-6	1–6 months	17.4% plasma-derived vaccine, Hevac-B;	IM	NA	92.2%
										83.5% recombinant vaccine, Engerix-B			
Oliveira L. C. *et al.*	2007	Randomized trials	a) 46.6 ± 10.9(alcoholics);b) 37.8 ± 9.7(non-alcoholics)	Healthy adults	Portugal	60/0	NA	0-1-6	1 month	20 μg recombinant vaccine, Euvax-B	IM	a) 511 ± 448 IU/L (alcoholics);b) 696 ± 410 IU/L (non-alcoholics)	a) 50% (alcoholics);b) 52.5% (non-alcoholics)
Wolters B. *et al.*	2009	a) Prospective cohort b) Retrospective cohort	a) Mean: 38.9 range: 18–79 b) Mean: 39.9 range: 16–75	Healthy adults	German	a) 109/65 b) 133/115	a) 25.5 ± 4.8 b) 24.4 ± 3.8	0-1-6	1–2 months	Twinrix	NA	1430 IU/L	88.7%
Kevorkyan A. K. *et al.*	2011	Retrospective cohort	40.3 ± 2.6	Health care workers	Bulgaria	13/57	NA	0-1-6	1–2 months	20 μg recombinant vaccine, Hepavax Gen	NA	NA	92.8%
Sheffield J. S. *et al.*	2011	Prospective cohort	25.3 ± 5.2	Pregnant women	USA	0/168	a) 26(responder);b) 36(non-responder)	0-1-4	5–6 months	recombinant vaccine, Recombivax HB	IM	NA	90.0%
De Schryver A. *et al.*	2011	Randomized trials	a) 41.4 ± 10.4 b) 42.5 ± 9.8	Healthy volunteers	Belgium	310/61	a) 26.1 ± 5.0 b) 26.6 ± 4.6	a) 0-1-6;b) 0-1-12	1 month	20 μg recombinant vaccine, Twinrix	IM	a) 1900.6 IU/L (0-1-12); b) 749.0 IU/L (0-1-6)	a) 95.6% (0-1-12);b) 97.1% (0-1-6)
Tohme R. A. *et al.*	2011	Retrospective cohort	82.2 ± 14.2 range: 45–102	Older adults	USA	7/25	25.4 ± 4.6	0-1-4	80–90 days	20 μg recombinant vaccine, Engerix-B	IM	4.8 IU/L	33.3%
Ren J. J. *et al.*	2012	Retrospective cohort	a) 32.45 ± 0.66 b) 33.69 ± 0.70 c) 31.71 ± 0.69 d) 32.20 ± 1.07 range: 16–49	Healthy adults	China	a) 242/351; b) 182/283; c) 246/333; d) 101/134	NA	0-1-6	1 month	10 μg recombinant vaccine producted by 4 different manufacturers	IM	a) 177.28 IU/L (Kangtai);b) 473.23 IU/L (Dalian HTB);c) 246.13 IU/L (GeneTech BP);d) 332.20 IU/L (GlaxoSmithKline)	a) 81.67% (Kangtai);b) 95.05% (Dalian HTB);c) 89.64% (GeneTech BP);d) 86.81% (GlaxoSmithKline)
Williams R. E. *et al.*	2012	Retrospective cohort	Median: 79.5 range 45–101	Older adults	USA	39/47	NA	0-1-6	1–2 months	1 mldose recombinant vaccine, Twinrix	IM	NA	34.0%
Chathuranga L. S. *et al.*	2013	Retrospective cohort	NA	Health care workers	Sri Lanka	190/152	NA	NA	2 months-14 years	NA	NA	NA	92.1%
Bender T. J. *et al.*	2014	Retrospective cohort	Median: 60 range: 46–86	Adults with assisted living facilities	USA	17/10	NA	0-1-7	1–2 months	1 mldose recombinant vaccine, Twinrix	IM	91.7 IU/L	81.0%
Thomas R. J. *et al.*	2015	Retrospective cohort	16–50	Health care workers	India	148/306	NA	0-1-6	1 month	20 μg recombinant vaccine, GeneVac-B	IM	NA	98.9%
Nashibi R. et al.*	2015	Retrospective cohort	31.9 ± 18.1 range: 20–55	Health care workers	Iran	43/196	a) 31.6 ± 7.5(responder);b) 33.4 ± 5.6(non-responder)	NA	1–6 months	NA	NA	NA	94.1%
a) Yao J. *et al.*	2015	Randomized trials	a) 32.75 ± 7.93 b) 33.31 ± 7.71 c) 33.16 ± 8.00	Healthy adults	China	a) 354/519; b) 338/523; c) 259/445	NA	a) 0-1-3; b) 0-1-6; c) 0-1-12	12 months	10 ug recombinant vaccine	IM	a) 213.16 IU/L (0-1-3); b) 432.58 IU/L (0-1-6); c) 451.47 IU/L (0-1-12)	a) 100% (0-1-3);b) 99.9% (0-1-6);c) 97.9% (0-1-12)
b) Yao J. *et al.*	2015	Randomized trials	a) Median: 30.23 range: 20.01–39.76 b) Median: 29.42 range: 20.01–39.92 c) Median: 30.25 range: 20.10–39.98	Seronegative adults	China	a) 100/149; b) 111/118; c) 84/124	NA	a) 0-1-3;b) 0-1-6;c) 0-1-12	1 month	10 ug recombinant vaccine	IM	a) 61.19 IU/L (0-1-3);b) 214.04 IU/L (0-1-6); c) 345.78 IU/L (0-1-12)	a) 83.9% (0-1-3);b) 88.2% (0-1-6);c) 94.2% (0-1-12)

NA: not available; IM: intramuscular; ID: intradermal.

*This article was regarded cross-sectional as cohort study.

**Table 2:** The absolute and relative risk of non-response to HBV vaccine by subgroup and evidence quality grading*.

Comparator	Intervention	Illustrative comparative risks*(per 1000, 95% CI)		Relative risk of non-response (95% CI)	Number of Participants (studies)	Quality of the evidence (GRADE)
		Assumed risk with comparator	Corresponding risk with intervention			
Age < 40	Age ≥ 40	105	195 (163 to 233)	1.85 (1.55 to 2.21)	10233 (19 studies)	⊕⊕⊕⊕ high
Age < 30	Age ≥ 30	58	99 (81 to 121)	1.72 (1.41 to 2.1)	5372 (13 studies)	⊕⊕⊕⊝ moderate
Age < 60	Age ≥ 60	284	370 (287 to 478)	1.30 (1.01 to 1.68)	480 (5 studies)	⊕⊕⊕⊝ moderate
Female	Male	124	176 (149 to 209)	1.42 (1.2 to 1.68)	10118 (20 studies)	⊕⊕⊕⊕ high
BMI < 25	BMI ≥ 25	125	186 (134 to 255)	1.48 (1.07 to 2.03)	5807 (10 studies)	⊕⊕⊕⊝ moderate
Non-smoker	Smoker	132	195 (152 to 248)	1.47 (1.15 to 1.87)	6935 (13 studies)	⊕⊕⊕⊕ high
Non-alcoholic	Alcoholic	50	43 (29 to 63)	0.86 (0.58 to 1.26)	2381 (5 studies)	⊕⊕⊕⊝ moderate
Healthy	Concomitant diseases	100	140 (104 to 187)	1.39 (1.04 to 1.86)	4386 (12 studies)	⊕⊕⊕⊕ high
Vaccine at 0-1-6 months	Vaccine at 0-1-12 months	32	45 (12 to 192)	1.39 (0.41 to 4.67)	2433 (4 studies)	⊕⊝⊝⊝ very low

GRADE: Grading of Recommendations, Assessment, Development and Evaluation.

*The results presented in the Table 2 were built around the assumption of a consistent relative effect. The implications of this effect for populations were considered at different baseline risks. Based on the assumed risks, corresponding risks after an intervention were calculated using the meta-analytic risk ratio.

## References

[b1] Lavanchy D. Hepatitis B virus epidemiology, disease burden, treatment, and current and emerging prevention and control measures. Journal of viral hepatitis 11, 97–107 (2004). 14996343 10.1046/j.1365-2893.2003.00487.x

[b2] European Centre for Disease Prevention and Control. Hepatitis B and C in the EU neighbourhood: prevalence, burden of disease and screening policies. European Centre for Disease Prevention and Control. 56 p. doi: 10.2900/30933 (Stockholm, 2010).

[b3] Hahne S. J. *et al.* Infection with hepatitis B and C virus in Europe: a systematic review of prevalence and cost-effectiveness of screening. BMC infectious diseases 13, 181, doi: 10.1186/1471-2334-13-181 (2013). 23597411 PMC3716892

[b4] Keck J. W. *et al.* Hepatitis B virus antibody levels 7 to 9 years after booster vaccination in Alaska native persons. Clinical and vaccine immunology: CVI 21, 1339–1342, doi: 10.1128/CVI.00263-14 (2014). 25056363 PMC4178570

[b5] Bruce M. G. *et al.* Antibody Levels and Protection After Hepatitis B Vaccine: Results of a 30-Year Follow-up Study and Response to a Booster Dose. The Journal of infectious diseases, doi: 10.1093/infdis/jiv748 (2016). 26802139

[b6] Yang S. G. *et al.* Effectiveness of HBV vaccination in infants and prediction of HBV prevalence trend under new vaccination plan: findings of a large-scale investigation. PloS one 7, e47808, doi: 10.1371/journal.pone.0047808 (2012). 23094094 PMC3477110

[b7] Su F. H. *et al.* Hepatitis B seroprevalence and anamnestic response amongst Taiwanese young adults with full vaccination in infancy, 20 years subsequent to national hepatitis B vaccination. Vaccine 25, 8085–8090, doi: 10.1016/j.vaccine.2007.09.013 (2007). 17920732

[b8] Melhem N. M., Rahhal N., Charide R., Kreidieh K. & El-Khatib R. Human immunodeficiency virus and viral hepatitis among high-risk groups: Understanding the knowledge gap in the Middle East and North Africa Region. World journal of hepatology 7, 2619–2630, doi: 10.4254/wjh.v7.i25.2619 (2015). 26557955 PMC4635148

[b9] McGregor I. A. Health and Communicable Disease in a Rural African Environment. Oikos 27, 180–192, doi: 10.2307/3543897 (1976).

[b10] Peto T. J. *et al.* Efficacy and effectiveness of infant vaccination against chronic hepatitis B in the Gambia Hepatitis Intervention Study (1986–90) and in the nationwide immunisation program. BMC infectious diseases 14, 7, doi: 10.1186/1471-2334-14-7 (2014). 24397793 PMC3898092

[b11] Fisman D. N., Agrawal D. & Leder K. The effect of age on immunologic response to recombinant hepatitis B vaccine: a meta-analysis. Clinical infectious diseases: an official publication of the Infectious Diseases Society of America 35, 1368–1375, doi: 10.1086/344271 (2002). 12439800

[b12] Zajac B. A., West D. J., McAleer W. J. & Scolnick E. M. Overview of clinical studies with hepatitis B vaccine made by recombinant DNA. The Journal of infection 13 Suppl A, 39–45 (1986). 2943814 10.1016/s0163-4453(86)92668-x

[b13] Jilg W., Schmidt M. & Deinhardt F. Vaccination against hepatitis B: comparison of three different vaccination schedules. The Journal of infectious diseases 160, 766–769 (1989). 2530289 10.1093/infdis/160.5.766

[b14] Morris C. A., Oliver P. R., Reynolds F. & Selkon J. B. Intradermal hepatitis B immunization with yeast-derived vaccine: serological response by sex and age. Epidemiology and infection 103, 387–394 (1989). 2530104 10.1017/s0950268800030740 PMC2249500

[b15] Westmoreland D., Player V., Heap D. C. & Hammond A. Immunization against hepatitis B–what can we expect? Results of a survey of antibody response to immunization in persons 'at risk' of occupational exposure to hepatitis B. Epidemiology and infection 104, 499–509 (1990). 2140795 10.1017/s0950268800047506 PMC2271775

[b16] Guan R., Tay H. H., Choong H. L., Yap I. & Woo K. T. Hepatitis B vaccination in chronic renal failure patients undergoing haemodialysis: the immunogenicity of an increased dose of a recombinant DNA hepatitis B vaccine. Annals of the Academy of Medicine, Singapore 19, 793–797 (1990). 2151841

[b17] Dahl-Hansen E., Siebke J. C., Froland S. S. & Degre M. Immunogenicity of yeast-derived hepatitis B vaccine from two different producers. Epidemiology and infection 104, 143–149 (1990). 2137785 10.1017/s0950268800054625 PMC2271739

[b18] Dentico P. *et al.* Long-term immunogenicity safety and efficacy of a recombinant hepatitis B vaccine in healthy adults. European journal of epidemiology 8, 650–655 (1992). 1426164 10.1007/BF00145379

[b19] Roome A. J., Walsh S. J., Cartter M. L. & Hadler J. L. Hepatitis B vaccine responsiveness in Connecticut public safety personnel. Jama 270, 2931–2934 (1993). 8254852

[b20] McMaster K. R. 3rd, Roper J. K. & Carter J. B. Intradermal hepatitis B vaccination in a 300-bed primary care hospital: experience with a recombinant vaccine in a four-dose schedule. American journal of infection control 21, 283–288 (1993). 8122799 10.1016/0196-6553(93)90384-g

[b21] Jaiswal S. B., Salgia P. B., Sepaha A. G. & Chitnis D. S. Hepatitis B vaccine in patients on haemodialysis. Lancet 346, 317–318 (1995). 7630276 10.1016/s0140-6736(95)92208-3

[b22] Bock H. L. *et al.* Immunogenicity of a recombinant hepatitis B vaccine in adults. Archives of internal medicine 156, 2226–2231 (1996). 8885822

[b23] Averhoff F. *et al.* Immunogenicity of hepatitis B Vaccines. Implications for persons at occupational risk of hepatitis B virus infection. American journal of preventive medicine 15, 1–8 (1998). 9651632 10.1016/s0749-3797(98)00003-8

[b24] Cardell K., Fryden A. & Normann B. Intradermal hepatitis B vaccination in health care workers. Response rate and experiences from vaccination in clinical practise. Scandinavian journal of infectious diseases 31, 197–200 (1999). 10447332 10.1080/003655499750006272

[b25] Ingardia C. J., Kelley L., Steinfeld J. D. & Wax J. R. Hepatitis B vaccination in pregnancy: factors influencing efficacy. Obstetrics and gynecology 93, 983–986 (1999). 10362167 10.1016/s0029-7844(98)00563-8

[b26] Young M. D. *et al.* Adult hepatitis B vaccination using a novel triple antigen recombinant vaccine. Hepatology 34, 372–376, doi: 10.1053/jhep.2001.26167 (2001). 11481622

[b27] Wolters B., Junge U., Dziuba S. & Roggendorf M. Immunogenicity of combined hepatitis A and B vaccine in elderly persons. Vaccine 21, 3623–3628 (2003). 12922091 10.1016/s0264-410x(03)00399-2

[b28] Martins R. M. *et al.* Multicenter study on the immunogenicity and safety of two recombinant vaccines against hepatitis B. Memorias do Instituto Oswaldo Cruz 99, 865–871, doi: S0074-02762004000800014 (2004). 15761604 10.1590/s0074-02762004000800014

[b29] Yen Y. H. *et al.* Study of hepatitis B (HB) vaccine non-responsiveness among health care workers from an endemic area (Taiwan). Liver international: official journal of the International Association for the Study of the Liver 25, 1162–1168, doi: 10.1111/j.1478-3231.2005.01157.x (2005). 16343067

[b30] Panhotra B. R., Saxena A. K., Al-Hamrani H. A. & Al-Mulhim A. Compliance to hepatitis B vaccination and subsequent development of seroprotection among health care workers of a tertiary care center of Saudi Arabia. American journal of infection control 33, 144–150, doi: 10.1016/j.ajic.2005.01.002 (2005). 15798668

[b31] Kulkarni P. S. *et al.* Immunogenicity of a new, low-cost recombinant hepatitis B vaccine derived from Hansenula polymorpha in adults. Vaccine 24, 3457–3460, doi: 10.1016/j.vaccine.2006.02.008 (2006). 16530299

[b32] Estevez Z. C. *et al.* Immunogenicity and safety assessment of the Cuban recombinant hepatitis B vaccine in healthy adults. Biologicals: journal of the International Association of Biological Standardization 35, 115–122, doi: 10.1016/j.biologicals.2006.06.001 (2007). 17056272

[b33] Locquet C., Marande J. L., Choudat D. & Vidal-Trecan G. Hepatitis B vaccination in women healthcare workers: a seroepidemiological survey. European journal of epidemiology 22, 113–119, doi: 10.1007/s10654-006-9094-x (2007). 17295098

[b34] Sabido M., Gavalda L., Olona N. & Ramon J. M. Timing of hepatitis B vaccination: its effect on vaccine response in health care workers. Vaccine 25, 7568–7572, doi: 10.1016/j.vaccine.2007.08.025 (2007). 17870215

[b35] Oliveira L. C., Silva T. E. & Alves M. H. Response to hepatitis B vaccine in alcoholics without clinically evident liver cirrhosis. Arquivos de gastroenterologia 44, 195–200 (2007). 18060270 10.1590/s0004-28032007000300003

[b36] Wolters B. *et al.* Comparative evaluation of the immunogenicity of combined hepatitis A and B vaccine by a prospective and retrospective trial. Human vaccines 5, 248–253 (2009). 19276678 10.4161/hv.5.4.7051

[b37] Kevorkyan A. K. *et al.* Immune response and immunologic memory in medical personnel vaccinated with hepatitis B vaccine. Folia medica 53, 32–38 (2011). 22359980 10.2478/v10153-011-0054-1

[b38] Sheffield J. S. *et al.* Efficacy of an accelerated hepatitis B vaccination program during pregnancy. Obstetrics and gynecology 117, 1130–1135, doi: 10.1097/AOG.0b013e3182148efe (2011). 21508752

[b39] De Schryver A. *et al.* Comparative immunogenicity of two vaccination schedules of a combined hepatitis A and B vaccine in healthy volunteers. Journal of viral hepatitis 18, e5–10, doi: 10.1111/j.1365-2893.2010.01365.x (2011). 20735800

[b40] Tohme R. A. *et al.* Evaluation of hepatitis B vaccine immunogenicity among older adults during an outbreak response in assisted living facilities. Vaccine 29, 9316–9320, doi: 10.1016/j.vaccine.2011.10.011 (2011). 22015390 PMC4610903

[b41] Ren J. J. *et al.* Immunological effects of a 10-mug dose of domestic hepatitis B vaccine in adults. *Journal of Zhejiang University*. Science. B 13, 948–954, doi: 10.1631/jzus.B1200179 (2012). 23125088 PMC3494034

[b42] Williams R. E. *et al.* Hepatitis B vaccination of susceptible elderly residents of long term care facilities during a hepatitis B outbreak. Vaccine 30, 3147–3150, doi: 10.1016/j.vaccine.2012.02.078 (2012). 22421557

[b43] Chathuranga L. S., Noordeen F. & Abeykoon A. M. Immune response to hepatitis B vaccine in a group of health care workers in Sri Lanka. International journal of infectious diseases: IJID: official publication of the International Society for Infectious Diseases 17, e1078–1079, doi: 10.1016/j.ijid.2013.04.009 (2013). 23810225

[b44] Bender T. J. *et al.* Hepatitis B vaccine immunogenicity among adults vaccinated during an outbreak response in an assisted living facility–Virginia, 2010. Vaccine 32, 852–856, doi: 10.1016/j.vaccine.2013.12.018 (2014). 24370706 PMC5719870

[b45] Thomas R. J. *et al.* Prevalence of non-responsiveness to an indigenous recombinant hepatitis B vaccine: a study among South Indian health care workers in a tertiary hospital. Indian journal of medical microbiology 33 Suppl, 32–36, doi: 10.4103/0255-0857.150877 (2015). 25657153

[b46] Nashibi R. *et al.* Post-vaccination Immunity Against Hepatitis B Virus and Predictors for Non-responders Among Medical Staff. Jundishapur journal of microbiology 8, e19579, doi: 10.5812/jjm.19579 (2015). 25861435 PMC4385250

[b47] Yao J. *et al.* Optimal vaccination program for healthy adults in China. Human vaccines & immunotherapeutics, 0, doi: 10.1080/21645515.2015.1053674 (2015). PMC4635859 26158622

[b48] Yao J. *et al.* The response of hepatitis B vaccination on seronegative adults with different vaccination schedules. Human vaccines & immunotherapeutics 11, 1102–1107, doi: 10.4161/21645515.2014.985500 (2015). 25621975 PMC4514229

[b49] Guyatt G. *et al.* GRADE guidelines: 1. Introduction-GRADE evidence profiles and summary of findings tables. Journal of clinical epidemiology 64, 383–394, doi: 10.1016/j.jclinepi.2010.04.026 (2011). 21195583

[b50] Egger M., Davey S. G., Schneider M. & Minder C. Bias in meta-analysis detected by a simple, graphical test. BMJ. Bmj 315, 477–477 (1997). 9310563 10.1136/bmj.315.7109.629 PMC2127453

[b51] Zeeshan M. *et al.* Evaluation of immune response to Hepatitis B vaccine in health care workers at a tertiary care hospital in Pakistan: an observational prospective study. BMC infectious diseases 7, 120, doi: 10.1186/1471-2334-7-120 (2007). 17961205 PMC2228304

[b52] Sangfelt P., Uhnoo I., Reichard O. & Weiland O. A low-dose intradermal hepatitis B vaccine programme in health-care workers and students is highly effective and cost saving: a retrospective follow-up survey in the clinical setting. Scandinavian journal of gastroenterology 43, 465–472, doi: 10.1080/00365520701733806 (2008). 18365912

[b53] Kanda N. & Tamaki K. Estrogen enhances immunoglobulin production by human PBMCs. The Journal of allergy and clinical immunology 103, 282–288 (1999). 9949320 10.1016/s0091-6749(99)70503-8

[b54] Kanda N., Tsuchida T. & Tamaki K. Testosterone inhibits immunoglobulin production by human peripheral blood mononuclear cells. Clinical and experimental immunology 106, 410–415 (1996). 8918592 10.1046/j.1365-2249.1996.d01-842.x PMC2200579

[b55] Klein S. L., Marriott I. & Fish E. N. Sex-based differences in immune function and responses to vaccination. Transactions of the Royal Society of Tropical Medicine and Hygiene 109, 9–15, doi: 10.1093/trstmh/tru167 (2015). 25573105 PMC4447843

[b56] Saunders J. B., Davis M. & Williams R. Do women develop alcoholic liver disease more readily than men? British medical journal 282, 1140–1143 (1981). 6786474 10.1136/bmj.282.6270.1140 PMC1505078

[b57] Hohler T. *et al.* Differential genetic determination of immune responsiveness to hepatitis B surface antigen and to hepatitis A virus: a vaccination study in twins. Lancet 360, 991–995, doi: 10.1016/S0140-6736(02)11083-X (2002). 12383669

[b58] Mentzer A. J., O'Connor D., Pollard A. J. & Hill A. V. Searching for the human genetic factors standing in the way of universally effective vaccines. Philosophical transactions of the Royal Society of London. Series B, Biological sciences 370, doi: 10.1098/rstb.2014.0341 (2015). PMC4527396 25964463

